# The Effect of Substitution of Mn by Pd on the Structure and Thermomagnetic Properties of the Mn_1−x_Pd_x_CoGe Alloys (Where x = 0.03, 0.05, 0.07 and 0.1)

**DOI:** 10.3390/ma16155394

**Published:** 2023-07-31

**Authors:** Karolina Kutynia, Anna Przybył, Piotr Gębara

**Affiliations:** Department of Physics, Częstochowa University of Technology, Armii Krajowej 19, 42-200 Częstochowa, Poland; karolina.kutynia@pcz.pl (K.K.); anna.przybyl@pcz.pl (A.P.)

**Keywords:** X-ray diffraction, DSC measurements, microstructure, magnetocaloric effect

## Abstract

In the present paper, the influence of partial substitution of Mn by Pd on structure, thermomagnetic properties, and phase transitions in the MnCoGe alloys was investigated. The studies of phase constitution revealed an occurrence of the orthorhombic TiNiSi-type and hexagonal Ni_2_Ti- type phases. Deep analysis of the XRD pattern supported by the Rietveld analysis allowed us to notice the changes in lattice parameters and quantity of recognized phases depending on the Pd content. An increase of palladium in alloy composition at the expense of manganese induced a rise in the Curie temperature. The values of ΔS_M_ measured for the variation of external magnetic field ~5 T equaled 8.88, 23.99, 15.63, and 11.09 for Mn_0.97_Pd_0.03_CoGe, Mn_0.95_Pd_0.05_CoGe, Mn_0.93_Pd_0.07_CoGe, and Mn_0.9_Pd_0.1_CoGe alloy, respectively. The highest magnetic entropy change ΔS_M_ was observed for samples with Pd content x = 0.05 induced by magnetostructural transformation. The analysis of the *n* vs. *T* curves allowed confirmation of the XRD and DSC results of an occurrence of the first-order magnetostructural transition in Mn_0.95_Pd_0.05_CoGe and Mn_0.93_Pd_0.07_CoGe alloys samples.

## 1. Introduction

Nowadays, taking care of the natural environment is extremely important. The consumption of energy for heating/cooling devices increases every year. Traditional cooling techniques are based on compression-decompression processes of freon gases, which are very dangerous for the ozone layer and global warming. Chlorofluorocarbons or hydrochlorofluorocarbons should be drastically reduced in the future, according to the Montreal protocol. The efficiency of cooling based on gas compression-decompression processes achieves about 45%. Research on novel, more efficient cooling techniques is being conducted. The most attractive, among others, is the magnetocaloric effect (MCE), discovered by Warburg in 1881 [1]. The MCE is the cooling or heating of magnetic material in an external magnetic field, and its efficiency reaches the highest values of even 60%. The magnitudes describing this phenomenon are adiabatic temperature change (ΔT_ad_) or isothermal magnetic entropy change (ΔS_M_) [2]. Since the discovery of the giant MCE in Gd_5_Ge_2_Si_2_ by Pecharsky and Gschneidner Jr. in 1997 [2,3], an enormous number of papers have been published. The MCE is observed in all magnetic materials, as it is the result of the coupling of the magnetic field with the magnetic sublattice, which leads to a change in the magnetic part of the entropy of the solids. The isothermal magnetic entropy change is measured indirectly based on field dependencies of magnetization collected at a wide range of temperatures [2,3].

The Gd_5_Ge_2_Si_2_ alloys are very hard in preparation and contain relatively expensive gadolinium and germanium elements, which are rare-earth alloys covered by embargoes related to the political situation in the world. These reasons induce high material prices for an active magnetic regenerator in commercial refrigerators. Taking into account the development of novel magnetic materials and lowering the price, many compounds were investigated, i.e., manganites [4], La(Fe,Si)_13_-type alloys [5,6], amorphous alloys [7], and others.

Researchers have been interested in MnCoGe alloys, which belong to the MM’X group of alloys (where M or M’—transition metal and X—metaloid) and are characterized by excellent magnetocaloric properties [8,9,10]. They are formed into independent crystal structures as low-temperature orthorhombic TiNiSi-type (space group Pnma) and high-temperature hexagonal Ni_2_Ti (space group P6_3_/mmc). For many years, the MnCoGe- prototype alloys have been modified by the introduction vacancies [11], off-stoichiometry composition [12], or partial substitution of the content element by others, i.e., Ge by Si [13]; Mn by V [14], Cr [15] or Pd [16]; Co by Ni [17]. Such modifications induced magneto-structural transition, which improved the magnetothermal properties. Qian and coworkers in [18] proposed a selective substitution of Mn by Zr. Such careful modification of alloy composition resulted in magneto-structural transition accompanied by strengthening of magnetic exchange interactions. All of these effects allowed the achievement of excellent values of magnetic entropy change near to the room temperature and relatively weak magnetic fields. In our previous paper [19], we also studied the influence of the partial substitution of Mn by Zr. The results delivered by Qian et al. in [18] were partially confirmed. As shown in [16], the partial substitution of Mn by Pd improves the value of magnetic entropy change and raises the Curie temperature. According to that, a much deeper analysis of Pd substitution in the range <0, 0.1>, including microstructural studies, the differential scanning calorimetry, and its influence on magnetic properties, is naturally expected, which was performed in the present paper. 

Taking into account the results described in papers [16,18,19], we decided to investigate the influence of selective substitution of Mn by Pd on the structure and magnetocaloric properties in the Mn_1−x_Pd_x_CoGe (where x = 0.03, 0.05, 0.07, and 0.1).

## 2. Sample Preparation and Experimental Details

The series of ingot samples corresponding to the following compositions: Mn_0.97_Pd_0.03_CoGe, Mn_0.95_Pd_0.05_CoGe, Mn_0.93_Pd_0.07_CoGe, and Mn_0.9_Pd_0.1_CoGe were produced by the arc melting technique in an atmosphere of inert gas (Ar). During the process, the high-purity elements (3N) were used. Taking into account good homogeneity, the specimens were remelted five times. The X-ray diffraction studies were carried out using a Bruker D8 Advance diffractometer with CuKα radiation. Phase recognition and quantity analysis were supported by Bruker EVA 4.0 software and PowderCell 2.4 package for the Rietveld refinement [20]. The thermomagnetic properties (the Curie temperature and magnetic isotherms) were investigated using the Quantum Design Physical Properties Measuring System (PPMS) model 6000, working with a wide range of magnetic fields and temperatures. Calorimetric measurements were carried out on a differential scanning calorimeter DSC 214 Polyma produced by Netzsch (Selb, Germany) using a heating and cooling rate of 10 K/min. The microstructure was photographed using scanning electron microscopy (SEM) JEOL 6610 LV equipped with energy dispersive X-ray spectrometer (EDS).

## 3. Results and Discussion

**Figure 1:**
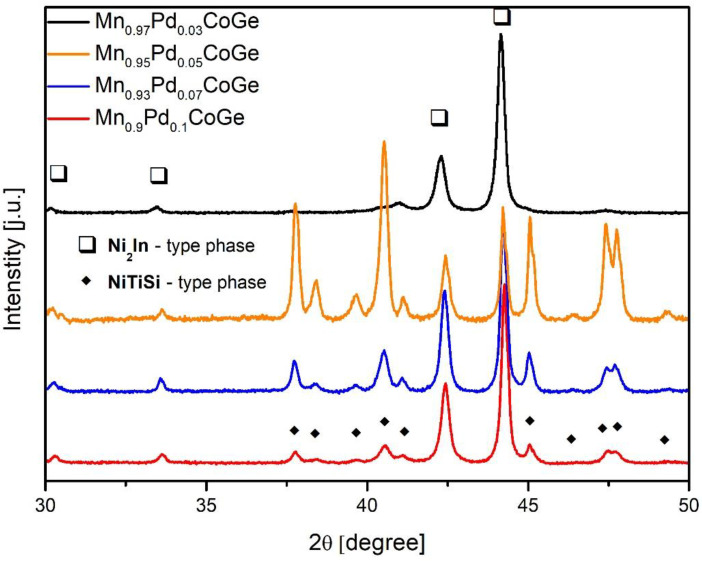
The X-ray diffraction patterns collected at room temperature for samples of produced alloys.

The ambient temperature XRD patterns of samples with different content of Pd were plotted in Figure 1. The analysis revealed the coexistence of two phases: the hexagonal Ni_2_In- type and orthorhombic NiTiSi- type, for all investigated specimens. A visible increase in the intensity of reflexes corresponding to the NiTiSi- type phase was observed. Moreover, the highest content of volume fraction of the NiTiSi- type phase with minor Ni_2_Ti-type phase for the Mn_0.95_Pd_0.05_CoSi alloy was detected. The calculations of the lattice constant of recognized phases showed a monotonic rise with an increase of Pd in a sample. Such effect is related to the different ionic radius of Pd (r_Pd_ = 1.37 Å) compared to much lower Mn radius (r_Mn_ = 1.18 Å). According to Bażela and coworkers in [21], the orthorhombic cell was treated as a distorted hexagonal cell. In such cases, the Pd atoms induce some distortions and promote crystallization hexagonal Ni_2_In-type phase. Careful examination of the X-ray diffraction patterns excluded an occurrence of some additional phases of impurities. The qualitative and quantitative analyses were strongly supported by the Rietveld refinement; the results are presented in Table 1.

**Figure 2:**
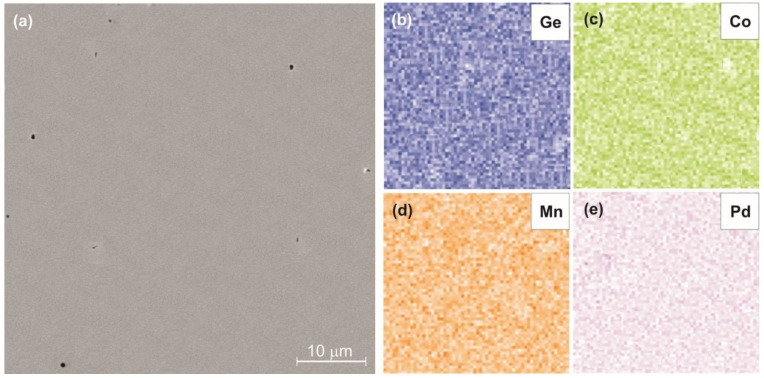
The SEM micrograph (**a**) and corresponding to it the EDS maps of the (**b**–**e**) Mn_0.95_Pd_0.05_CoGe alloy sample.

The homogeneity of the samples was studied using the SEM technique. The micrograph of the Mn_0.95_Pd_0.05_CoGe alloy sample is shown in Figure 2a. EDS maps, collected for observed microstructure, present homogenous distribution of constituent elements (Figure 2b–e). Moreover, the concentration of nominal composition Mn—31.67 at.%, Pd—1.67 at.%, Co—33.33 at.% and Ge—33.33 at.% corresponds well with measured by EDS Mn—31.45 ± 0.22 at.%, Pd—1.65 ± 0.18 at.%, Co—33.42 ± 0.25 at.%, and Ge—33.41 ± 0.36 at.%, respectively.

As it was reported by Johnson in [22], the structural transition from hexagonal to orthorhombic structure was called a martensitic-type diffusionless transition. Moreover, he showed some relations between the lattice parameters of these two phases, which were given as [22]:

(1) $$a_{orth}\approx c_{hex};\;b_{orth}\approx a_{hex};\;c_{orth}\approx \sqrt{3}a_{hex}$$

**Figure 3:**
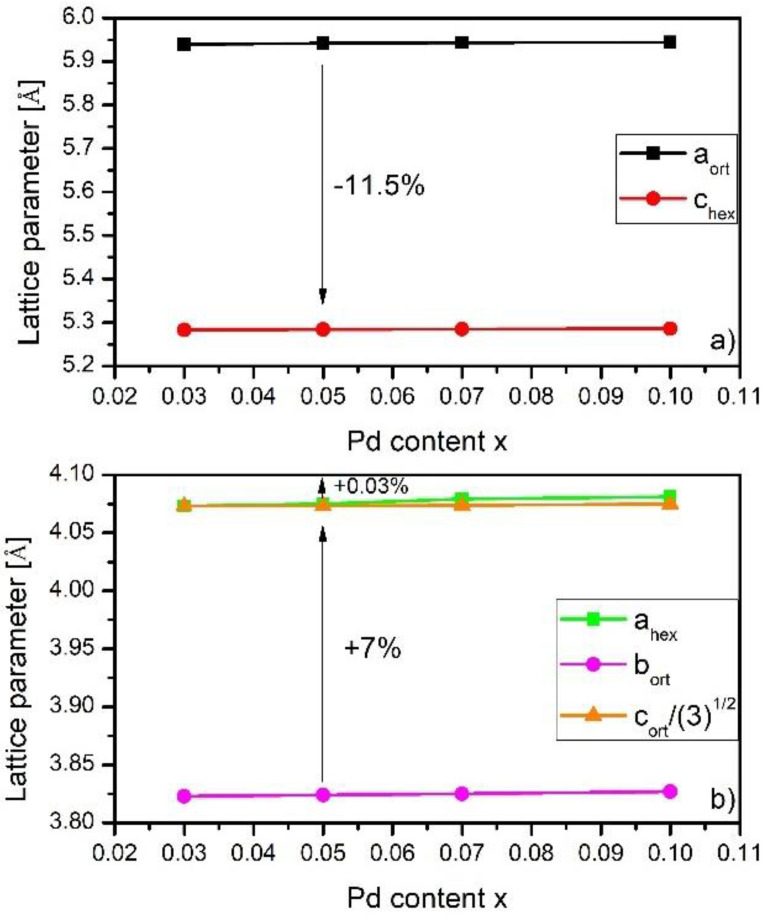
The Pd content x dependence of the unit cell parameters constructed for Mn_1−x_Pd_x_CoGe (where x = 0.03, 0.05, 0.07, and 0.1). Due to the symbol size being comparable to the error, the errors were not matched. (**a**) Pd content x vs. *a_ort_* and *c_hex_*, (**b**) Pd addition x vs. *a_hex_*, *b_ort_* and *c_ort_*/(3)^1/2^.

These relations were used to construct Pd content dependence of lattice parameters of the hexagonal Ni_2_In-type structure and orthorhombic NiTiSi-type structure for investigated samples presented in Figure 3. During the transformation from orthorhombic to hexagonal, the first cell contracts by 11.5% along the a-axis. However, the enlargements of the orthorhombic structure by 7% and 0.03% along the b and c axes were noticed. Such behavior is expected in magnetocaloric materials because magnetostructural transformation leads to an increase in the total entropy of the material, even about 90% [23]. 

**Figure 4:**
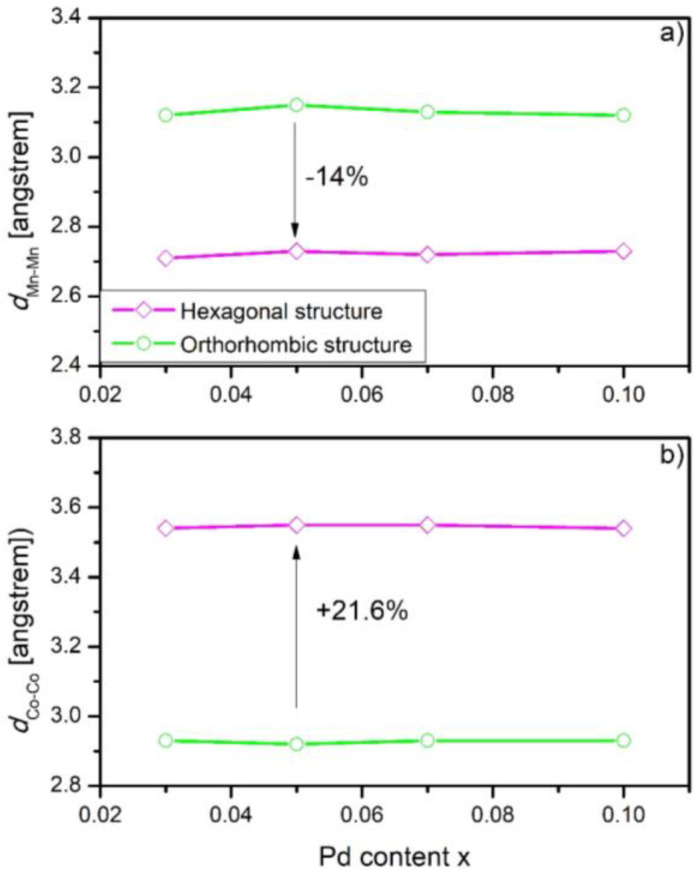
The Pd content x dependences of interatomic distances calculated for the Mn_1−x_Pd_x_CoGe samples (**a**) Mn-Mn distances and (**b**) Co-Co distances. The errors were not marked due to being smaller than the symbol size.

As shown by Qian and coworkers in Reference [18], the nearest interatomic distances could manifest some variations depending on the Zr content. Taking into account these results, the Mn-Mn and Co-Co distances were calculated and presented as Pd content dependence (Figure 4). Significantly shorter Mn-Mn distances were observed, while the Co-Co distances increased visibly. The results are similar to the data delivered in [18]. Such variations of distances of magnetic elements significantly influenced magnetic exchange interactions, which resulted in strong coupling between the magnetic and structural transitions.

**Figure 5:**
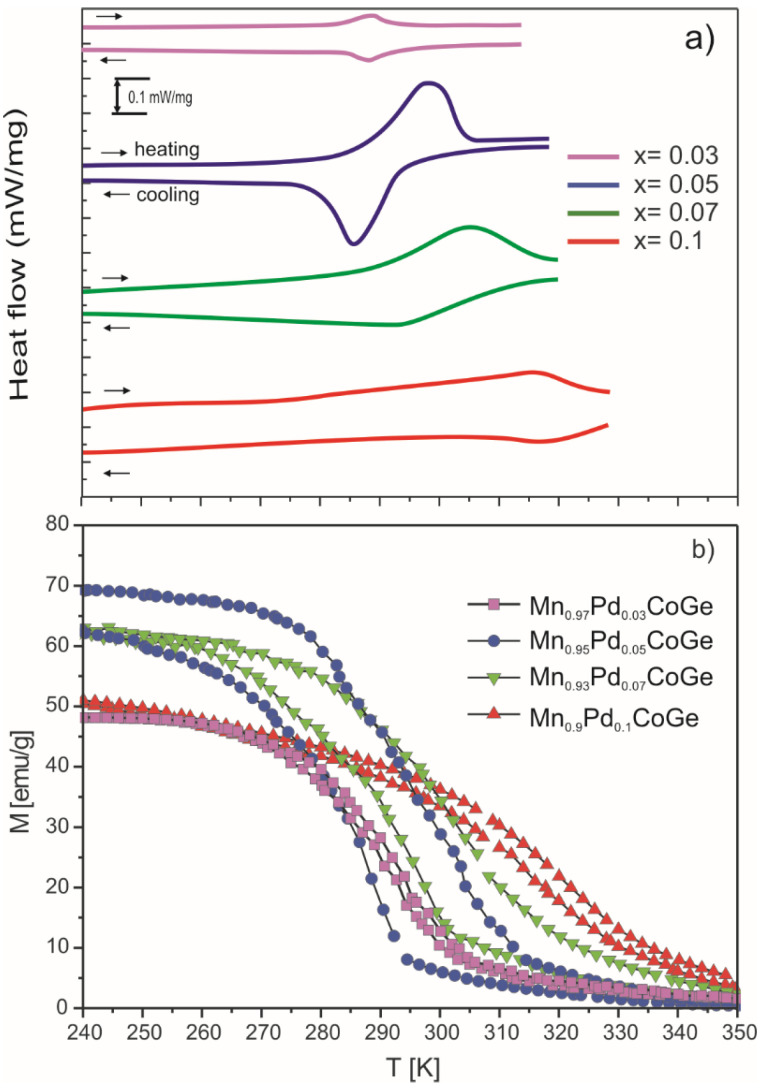
The DSC curves (**a**) and temperature dependences of magnetization (field cooling regime at Δ(μ_0_H) = 0.1 T) (**b**) collected for the studied specimens.

In order to confirm the results of the XRD analysis and determination of temperatures of structural and magnetic transitions, the DSC measurements were conducted (Figure 5a). The lambda-type peaks were detected for samples with the lowest (x = 0.03) and the highest (x = 0.1) Pd content in the vicinity of 290 and 315 K, respectively. These temperatures are in agreement with the Curie temperature manifested by *M* vs. *T* curves collected for appropriate specimens. The lambda peaks were not observed for two of the samples, while the magnetostructural transition was detected. Similar results were observed for (Mn,Zr)CoGe samples by Qian et al. in [18]. The Mn_0.95_Pd_0.05_CoGe alloy sample shows the structural transition from the paramagnetic hexagonal to a ferromagnetic orthorhombic structure at 300 K. Moreover, a visible temperature hysteresis was observed, which was confirmed by thermomagnetic measurements (Figure 5b).

The M vs. T dependences measured in the external magnetic field up to 0.1 T (in field cooling regime) for all samples are presented in Figure 5b. The thermal hysteresis is visible in all studied samples, which could suggest an occurrence of first-order phase transition in the produced materials [24]. A first derivative of M(T) dependences allowed us to obtain the values of the Curie temperature. The minimum of the dM(T)/dT curves made it possible to estimate the Curie point, which equaled 294 ± 1 K, 298 ± 1 K, 307 ± 1 K, and 318 ± 1 K for the concentration of Pd content x = 0.03, 0.05, 0.07, and 0.1, respectively. Moreover, the gradual increase of the T_C_, was expected, based on results delivered in [18]. Such a rise in the Curie point could be related to an increase in the lattice constant with an increase in Pd content, which was confirmed by the XRD studies. As mentioned, the Pd atoms are larger than the Mn atoms, and the structure expands (Table 1). It could induce strengthening interactions between Mn—Mn and Mn—Co atoms.

Further studies concerning thermomagnetic properties were conducted in order to show the magnetocaloric properties. They were investigated indirectly based on magnetic isotherms measured in a wide range of temperatures. Measured curves allowed for calculations of magnetic entropy change Δ*S_M_* according to the following relation [25]:

(2) $$\Delta S_{M}(T,\Delta H)=\mu_{0}\int_{0}^{H}{\left(\frac{\partial M(T,H)}{\partial T}\right)}_{H}dH,$$

where *μ*_0_, *H*, *M*, and *T* represent the magnetic permeability of the vacuum, the strength of the magnetic field, magnetization, and temperature, respectively.

Due to the fact that discrete data were used for the calculation, the Relation (2) was realized using the following algorithm [26]:

(3) $$\Delta S_{M}(\frac{T_{i}+T_{i+1}}{2})\approx \frac{1}{T_{i+1}-T_{i}}\left[\int_{0}^{B_{\max}}M(T_{i+1},B)dB-\int_{0}^{B_{\max}}M(T_{i},B)dB\right]$$

where *B* represents the induction of the magnetic field, related to equation *B = μ*_0_*H.*

**Figure 6:**
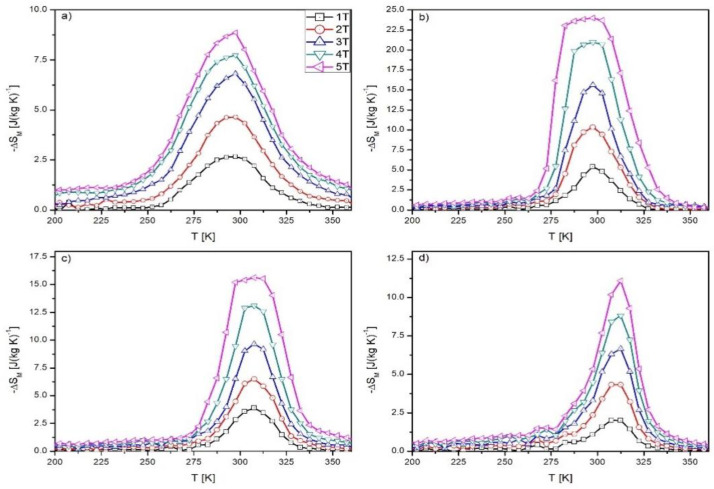
The *ΔS_M_* vs. *T* curves revealed for Mn_0.97_Pd_0.03_CoGe (**a**), Mn_0.95_Pd_0.05_CoGe (**b**), Mn_0.93_Pd_0.07_CoGe (**c**), and Mn_0.9_Pd_0.1_CoGe (**d**) specimens.

The temperature dependences of the magnetic entropy change determined for all investigated samples are shown in Figure 6. Symmetrical (caret) shapes of Δ*S_M_*(*T*) dependences were observed for samples Mn_0.97_Pd_0.03_CoGe and Mn_0.9_Pd_0.1_CoGe alloys, which suggests an occurrence of second-order phase transition [27]. Otherwise, an asymmetric shape was observed for Mn_0.95_Pd_0.05_CoGe and Mn_0.93_Pd_0.07_CoGe alloys, which is indirect proof of first-order phase transition and confirms DSC observations. The values of magnetic entropy change Δ*S_M_* measured under the change of external magnetic field ~5 T equaled 8.88, 23.99, 15.63, and 11.09 for Mn_0.97_Pd_0.03_CoGe, Mn_0.95_Pd_0.05_CoGe, Mn_0.93_Pd_0.07_CoGe, and Mn_0.9_Pd_0.1_CoGe alloy, respectively. Taking into account the value of Δ*S_M_* calculated for the base MnCoGe alloy studied in [18], in the present case, they reached higher values. Moreover, similar to results delivered in previous work [19] concerning the selective substitution of Mn by Zr, the magnetic entropy change was the highest for the Pd content x = 0.05. Further increase of Pd content involved the reduction of magnetic entropy change value; however, even for rest additions (x = 0.07 and 0.1) was noteworthy. Such a high increase of the magnetic entropy change (for x = 0.05 sample) was caused by the magnetostructural transition visible in the DSC curve measured for this alloy. Revealed values are higher than the values delivered in a previous paper [19] concerning (Mn,Zr)CoGe alloys; however, they are comparable or slightly higher than the values described in [12,13,15,28,29,30]. Relatively large magnetic entropy change is probably caused by the magnetostructural phase transition, similar to that observed in [18,19] for Zr-doped MnCoGe alloys. It is realized as a structural reconfiguration, similar to the order–disorder setting of magnetic moments. A combination of lattice rearrangement and magnetic transition at *T_C_* may induce a giant magnetocaloric effect because the total entropy of magnetic material is a sum of magnetic, lattice, and electronic contributions. This last term could be omitted because studied materials do not manifest itinerant electron metamagnetic transition. A similar effect was observed by Qian et al. in [18] during studies of (Mn,Zr)CoGe alloys.

Taking into account the practical utility of fabricated alloys in household appliances, the refrigeration capacity (*RC*) was studied. The *RC* values were calculated based on *ΔS_M_* vs. *T* curves using the following equation [31]:

(4) $$RC(\delta T,H_{MAX})=\int_{T_{cold}}^{{}_{T_{hot}}}\Delta S_{M}(T,H_{MAX})dT$$

where *RC* and *H_MAX_* represent refrigerant (cooling) capacity and maximum amplitude of external magnetic field change, δ*T* = *T_hot_* − *T_cold_* is related to the temperature range of the thermodynamic cycle (in practical calculations, the full width at half maximum of magnetic entropy change peak). 

The calculated values of magnetic entropy change and cooling capacity for several values of the external magnetic field are collected in Table 2. The highest value of refrigeration capacity was calculated for the Mn_0.95_Pd_0.05_CoGe alloy sample, which is comparable with the values delivered for Gd-based amorphous alloys by Pierunek et al. in [32]. Moreover, the delivered values are higher than the ones revealed for samples with Zr addition [19].

Law and coworkers in [33] proposed a relatively fast technique for investigation and order of phase transition. The Law–Franco method is based on the phenomenological *B* vs. *H* curve [29], which could be written in the following form:

(5) $$\Delta S_{M}=C\cdot {\left(B_{MAX}\right)}^{n}$$

where *C* is the proportionality constant depending on temperature and *n* is the exponent strongly dependent on the magnetic ordering of the specimen. 

Franco and coworkers in [34] proposed that the calculations of the exponent *n* are possible based on the modification of Equation (5) in the following relation:

(6) $$n=\frac{dln|{∆S}_{M}|}{dln|H|}$$

A more simple way to make known the *n* exponent was proposed in [35], and Equation (5) was rewritten in the present form:

(7) $$\ln\Delta S_{M}=\ln C+n\ln B_{MAX}$$

As shown in [34], the exponent *n* is hardly dependent on the magnetic ordering of the material. Assuming that the studied material obeys the Curie–Weiss law, the exponent should equal 1 and 2 in the ferromagnetic and paramagnetic states, respectively. Its value at *T_C_* is strongly related to critical exponents and could be written in the following form:

(8) $$n=1+\frac{1}{\delta \left(1-\frac{1}{\beta }\right)}$$

where *β* and *δ* are critical exponents in the vicinity of the critical point (i.e., *T_C_*).

Recently, Moreno-Ramirez et al. in [36] presented more precise conditions of the Law–Franco method adapted for the investigation of first-order phase transition. They showed that during phase transition, exponent *n* reaches values much higher than 2. Moreover, the *n*(*T_C_*) is lower than 0.4. Similar results were observed for Gd_5_Si_2_Ge_2_- type alloys studied in [37]. 

**Figure 7:**
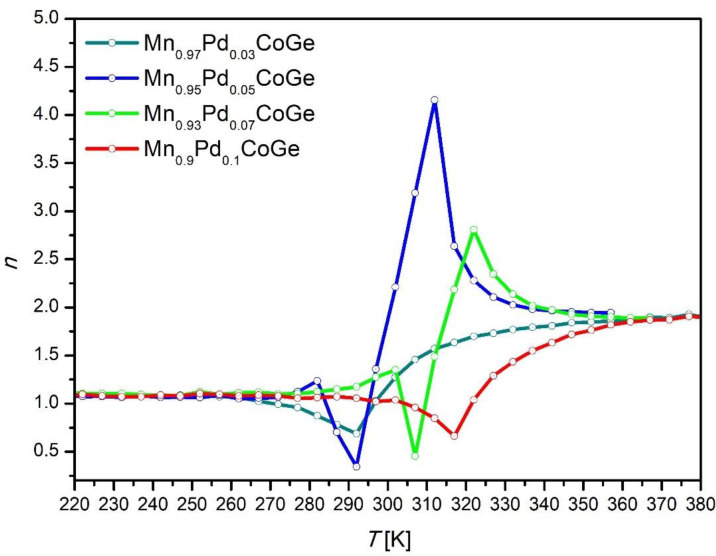
Temperature evolutions of exponent *n* revealed for all studied specimens.

The *n* vs. *T* curves for all investigated alloy samples are collected in Figure 7. The shape of the *n* vs. *T* curves constructed for the Mn_0.97_Pd_0.03_CoGe and Mn_0.9_Pd_0.1_CoGe alloys is characteristic of the second-order phase transition. In the case of rest two samples, an evident first-order phase transition is detected. Moreover, close to the Curie point, a characteristic hump is observed, which is typical for structural transformation, similar to the results described in [33,37]. The values of the exponent *n* revealed at the Curie point of Mn_0.95_Pd_0.05_CoGe and Mn_0.93_Pd_0.07_CoGe were 0.34 and 0.45, respectively. Such values on exponent *n* confirmed the first order phase transition in these two alloys, taking into account the results delivered in [33,36,37].

## 4. Conclusions

The studies conducted in the present paper were focused on the influence of the partial substitution of Mn by Pd on the structure, thermomagnetic properties, and phase transitions in the MnCoGe alloys. The XRD investigation showed an occurrence of two phases in all investigated samples, the orthorhombic TiNiSi-type phase and hexagonal Ni_2_In- type phases with different content depending on the Pd addition. Deep analysis of the X-ray diffraction patterns assisted by the Rietveld refinement revealed a structure transformation. A rise of palladium content in the alloy at the expense of manganese induced an increase in the Curie temperature. The DSC studies showed lambda peaks corresponding to the Curie temperature and characteristic peaks associated with the magnetostructural transition. The maximum value of magnetic entropy change was observed for the sample with a Pd content x = 0.05, and it was induced by magnetostructural first-order phase transition. This transition was manifested by the enormous changes in the interatomic distances between the magnetic exchange interactions. An occurrence of lattice transformation and magnetic transition involved giant magnetic entropy change. Moreover, for the Mn_0.93_Pd_0.07_CoGe alloy sample, the same type of transition was detected, which resulted in relatively good magnetocaloric properties. In the case of Mn_0.97_Pd_0.03_CoGe and Mn_0.9_Pd_0.1_CoGe alloy samples, a relatively high value of the ΔS_M_ was calculated. The presence of magnetostructural first-order phase transition in the Mn_0.95_Pd_0.05_CoGe and Mn_0.93_Pd_0.07_CoGe alloy samples was confirmed by careful analysis of the *n* vs. *T* curves. 

## Figures and Tables

**Table 1:** The data delivered by the Rietveld analysis for investigated Mn_1−x_Pd_x_CoGe alloys samples.

Alloy	Recognized Phases	Lattice Constant [Å] ± 0.001	Volume Fraction [%]
Mn_0.97_Pd_0.03_CoGe	hex Ni_2_Ti-type	a = 4.073	93
		c = 5.283	
	ort NiTiSi-type	a = 5.939	7
		b = 3.823	
		c = 7.053	
Mn_0.95_Pd_0.05_CoGe	hex Ni_2_Ti-type	a = 4.075	34
		c = 5.285	
	ort NiTiSi-type	a = 5.942	66
		b = 3.824	
		c = 7.055	
Mn_0.93_Pd_0.07_CoGe	hex Ni_2_Ti-type	a = 4.079	52
		c = 5.285	
	ort NiTiSi-type	a = 5.943	48
		b = 3.825	
		c = 7.056	
Mn_0.9_Pd_0.1_CoGe	hex Ni_2_Ti-type	a = 4.081	45
		c = 5.286	
	ort NiTiSi-type	a = 5.944	55
		b = 3.827	
		c = 7.058	

**Table 2:** Thermomagnetic data (Δ*S_M_* and cooling capacity *RC*) for Mn_0.97_Pd_0.03_CoGe, Mn_0.95_Pd_0.05_CoGe, Mn_0.93_Pd_0.07_CoGe, and Mn_0.9_Pd_0.1_CoGe alloys.

Sample	Magnetic Field Change Δ(μ_0_H) [T]	Magnetic Entropy Change Δ*S_M_* [J (kg K)^−1^]	Cooling Capacity RC [J kg^−1^]
Mn_0.97_Pd_0.03_CoGe	1	2.67	90
	2	4.64	154
	3	6.82	267
	4	7.75	317
	5	8.88	402
Mn_0.95_Pd_0.05_CoGe	1	5.41	104
	2	10.37	249
	3	15.62	365
	4	20.98	499
	5	23.99	646
Mn_0.93_Pd_0.07_CoGe	1	3.91	93
	2	6.50	165
	3	9.65	225
	4	13.10	320
	5	15.63	463
Mn_0.9_Pd_0.1_CoGe	1	2.02	39
	2	4.33	86
	3	6.67	131
	4	8.82	209
	5	11.09	238

## Data Availability

The data presented in this study are available on request from the corresponding author.
